# Differences in diabetes prevalence and inequalities in disease management and glycaemic control by immigrant status: a population-based study (Italy)

**DOI:** 10.1186/s12889-015-1403-4

**Published:** 2015-02-06

**Authors:** Paola Ballotari, Stefania Caroli, Francesca Ferrari, Gabriele Romani, Greci Marina, Antonio Chiarenza, Valeria Manicardi, Paolo Giorgi Rossi

**Affiliations:** Servizio Interaziendale di Epidemiologia, Local Health Authority, Via Amendola 2, Reggio Emilia, Italy; IRCCS, Arcispedale Santa Maria Nuova, Reggio Emilia, Italy; Primary Health Care, Local Health Authority, Reggio Emilia, Italy; Research and Innovation Unit, Local Health Authority, Reggio Emilia, Italy; Department of Internal Medicine, Hospital of Montecchio, Local Health Authority, Reggio Emilia, Italy

**Keywords:** Diabetes, Difference, Inequality, Immigrants, Prevalence, Disease management

## Abstract

**Background:**

The diabetes prevalence increases at an alarming rate around the world and understanding disparities in occurrence, care management, and health outcomes may be a starting point towards achieving more effective strategies to prevent and manage it. The aims of this study are to compare immigrants and Italians in terms of the differences in diabetes prevalence and to evaluate inequalities in disease management and glycaemic control by using information included in Reggio Emilia diabetes register.

**Methods:**

We retrieved from the diabetes register subjects aged 20–74 on December 31^st^, 2009. Using citizenship, we created three main groups: Italy, High Developed Countries (HDC), and High Migration Pressure Countries (HMPC). These were split into sub-regions of origin. We calculated age-adjusted prevalence by gender and sub-region. Using logistic regression model, we analyzed the association between area of origin and following indicators: 1) not being in care of diabetes clinics; 2) not having glycated haemoglobin (HbA1c) test in 2010; 3) among those tested, having a HbA1c value > = 9% (75 mmol/mol).

**Results:**

We found 15,889 Italian and 1,295 HMPC citizens with diabetes. HMPC citizens had higher age-adjusted prevalence of diabetes than Italians (females 5.0% vs 3.6%; males 6.5% vs 5.5%). The excess was mostly due to a strong excess in immigrants from Southern Asia (females 9.7%, males 10.2%) and Northern Africa (females 9.3%, males 5.9%). HMPC citizens were cared for by diabetes clinics in a similar proportion than Italians (OR: 1.08; 95% CI: 0.93-1.25), but had a greater odds of not being tested for HbA1c (OR: 1.51; 95% CI: 1.34-1.71), as well as of having HbA1c values equal to or over 9% (OR: 2.06; 95% CI: 1.80-3.14). The outcomes were poorer in HMPC females for the first two outcomes, while there was no difference for the HbA1c values (Wald test for heterogeneity p = 0.0850; p = 0.0156; p = 0.6635, respectively).

**Conclusions:**

Our findings highlight the need for gender-oriented actions for prevention and early diagnosis of the diabetes to contrast the higher risk in Northern Africans and Southern Asians. Further studies are required to determine whether the protocols in use are adequate for different immigrant groups.

**Electronic supplementary material:**

The online version of this article (doi:10.1186/s12889-015-1403-4) contains supplementary material, which is available to authorized users.

## Background

Diabetes is a major health problem that is increasing at an alarming rate around the world. In 2013, 382 million people had diabetes; this number is expected to rise to 592 million by 2035, potentially leading to major medical, social, and economic problems [1]. Understanding disparities in occurrence, care management, and health outcomes may be a starting point towards achieving more effective strategies to prevent and manage diabetes.

International studies provide strong evidence that ethnicity is an important determinant of diversity in the occurrence of diabetes [2-11]. In addition, studies have shown that immigrants with diabetes are undermanaged [12-14], and some ethnic groups have a lower probability of testing glycaemic index and reaching the recommended levels of glycated haemoglobin (HbA1c) [15-18]. As with other diseases, the relationship between diabetes and migration can be difficult to disentangle as the former is influenced by ethnic, socioeconomic, lifestyle, individual factors, and the latter by the country of origin, reasons for migration, age at arrival, and duration of stay in the host country.

In Italy, the foreign population has grown steadily over the years, from 0.6% of the resident population in 1991 to 7.0% in 2009 [19-21]. In Reggio Emilia (northern Italy) province, the percentage of immigrants was 12.3% at the end of 2009, one of the highest in Italy [20,21]. Further, the immigrant population is very heterogeneous, with people from more than 100 countries. The first three largest communities (i.e. Morocco, Albania, India) together account only for 36% of the foreigners.

The aims of our study are to compare immigrants and Italians in terms of the differences in diabetes prevalence and to evaluate inequalities in disease management and glycaemic control by using information included in Reggio Emilia diabetes register.

## Methods

### Setting and study population

Reggio Emilia is a province situated in Emilia-Romagna region, northern Italy. The health system is administered by the local health authority; the province is divided in six health districts. The local health authority of Reggio Emilia is a partner of the International Network of Health Promoting Hospitals & Health Services (HPH) and leader of the Task Force on Migrant Friendly and Culturally Competent Health Care (TF MFCCH). As consequence of this immigrant-sensitive strategy, the local health authority management has over the years encouraged studies aimed at monitoring immigrant health status and health services utilization.

For the present study we took into consideration all inhabitants in the Reggio Emilia province aged 20–74 on December 31^st^, 2009: 369,435 individuals. Of these, there were 17,195 subjects who had been diagnosed with diabetes on or before December 31^st^, 2009, were alive on that date, and were included in the Reggio Emilia diabetes register (accessed on November, 8^th^, 2013), achieving a prevalence of 4.6%. The methods applied to develop our disease register have been described elsewhere [22]. In brief, the register was created by deterministic linkage of six routinely collected data sources through a definite algorithm able to ascertain cases and to distinguish type of diabetes and model of care. The sources are: Hospital Discharge, Drug Dispensation, Biochemistry Laboratory, Disease-specific Exemption, Diabetes Outpatient Clinics, and Mortality databases. Women with gestational diabetes or women receiving treatment for polycystic ovarian syndrome were excluded.

There are 7 diabetes outpatient clinics in the Reggio Emilia province that provide specialized care for diabetes patients. Regional disease management guidelines [23] recommend that patients with Type 1 diabetes are always followed by diabetes outpatient clinic or paediatric unit. According to regional guidelines, newly diagnosed Type 2 diabetes patients are referred by their general practitioner (GP) to diabetes clinics for a preliminary evaluation. The specialist at diabetes clinic identifies those patients eligible for shared care management (in the case of well-controlled Type 2 diabetes) and those that should be cared for exclusively by a diabetes clinic (i.e. in the case of diabetes with chronic complications, insulin therapy, or unstable control of disease). Consequently, the patients not in care of diabetes clinics could either be in the care of their GP only or not cared for at all.

### Outcomes and other variables

According to the regional guidelines [23], we calculated two indicators of disease management and one indicator as intermediate outcome of care: 1) the percentage of people with diabetes who were not in care of diabetes clinics; 2) the percentage of those who had not at least one HbA1c test in 2010 (excluding those who had died or moved away in that year); 3) among those tested, the percentage with the most recent HbA1c value equal to 9% (75 mmol/mol) or over (marker of very poor glycaemic control according to regional guidelines [23,24]).

The results are presented by sex and citizenship, and grouped by geographical area of origin. First, we aggregated the citizenships into three main groups: Italy, High Developed Countries (HDC), and High Migration Pressure Countries (HMPC) [25]. Then, in accordance with the UN geoscheme [26], we split HMPC in sub-regions of provenience: Europe no HDC, Northern Africa, sub-Saharan Africa, Southern Asia, Rest of Asia, LA and the Caribbean. Annex 1 (Additional file 1) details country classification by area of origin, reporting for each country also the number of inhabitants, number of subjects with diabetes, and crude prevalence. Subjects with diabetes were dichotomized as “not treated” if no drug prescription was found in our databases in 2009–2010 or “treated” if anti-diabetic drug (insulin or oral hypoglycaemic) prescriptions were found.

### Statistical analyses

We calculated age-adjusted point prevalence rates of diabetes by sex and sub-region on December 31^st^, 2009. To perform direct age-standardization, we used the 2009 Reggio Emilia population in order to adjust for differences in population distribution across different world sub-regions.

Furthermore, we computed the mean age with confidence intervals and we reported the number and percentage of subjects not treated with anti-diabetic drugs, not in care of diabetes clinics, without HbA1c test in 2010, and, among those tested, with latest HbA1c assay > = 9% (75 mmol/mol).

Lastly, we used the multivariate logistic regression model to analyze the association between immigrant status and the outcomes defined above, using Italian citizenship as comparator and adjusting for age (kept as continous variable), sex, and treatment. In the analysis by sub-region, sex was included in the model as further covariate. To evaluate the interaction between main groups of citizenship and sex, a Wald test was applied. To investigate whether having a prescription of anti-diabetes drugs was a possible intermediate factor between the exposure variable “citizenship” and the outcome variable “having HbA1c > = 9% (75 mmol/mol)”, we show the results of logistic model with and without the treatment as covariate. A 5% significance level was used for all analyses. Analyses were performed using the STATA statistical package Version 11.

### Ethical approval

This is an observational study and data were collected retrospectively. The local health authority of Reggio Emilia was responsible for collecting and elaborating these data. The study was commissioned by the local health authority. The Reggio Emilia diabetes registry was approved by the provincial Ethical Committee in July 2014. According to Italian privacy law, no patient’s or parents’ consent is required for large retrospective population-based studies and when data are published only in aggregated form.

## Results

### Prevalence

In our register, we found 15,889 (92.4%) Italians, 11 citizens from HDC (0.1%), and 1,295 (7.5%) from HMPC with diabetes and aged 20–74. There were 15,151 (95.3%), 9 (81.8%), and 1,225 (94.6%) subjects with Type 2 diabetes, respectively. Foreigners were younger than Italians (Table 1), and the lowest age means were found among men and women from sub-Saharan Africa (F: mean 44.6 years, 95% CI: 41.4-47.9; M: mean 44.9 years, 95% CI: 43.2-46.7).

**Table 1:** **Characteristics of subjects with diabetes on December 31** ^**st**^ **, 2009: 20–74 years**

**Area of origin (n. of subject with diabetes)**	**Sex**	**Prevalence**			**Age**		**Not treated**		**Not in care of diabetes clinics**		Without HbA1c test in 2010*		**Among tested, HbA1c > =9%**	
		**Crude**	**Adjusted**	**95% CI**	**Mean**	**95% CI**	**N**	**%**	**N**	**%**	**N**	**%**	**N**	**%**
Italy (15,889)	F	4.1	3.6	3.5-3.7	62.0	61.7-62.2	1,423	21.9	1,455	22.3	1839	28.6	586	12.6
	M	5.8	5.5	5.4-5.6	61.2	61.0-61.4	1,982	21.1	2,083	22.2	2892	31.5	769	12.0
HDC (11)	F	2.1	2.2	0.4-4.0	51.8	43.7-60.0	1	14.3	1	14.3	2	33.3	1	25.0
	M	7.5	1.7	0.1-3.4	61.2	55.9-66.1	1	25.0	1	25.0	2	50.0	0	0.0
HMPC (1,295)	F	2.7	5.0	4.5-5.5	50.8	49.9-51.8	133	21.5	155	25.1	288	47.1	94	29.0
	M	3.1	6.5	5.8-7.1	48.6	47.8-49.4	117	17.3	128	18.9	285	42.5	104	26.9
Europe no HDC (281)	F	1.8	3.1	2.5-3.7	54.9	53.2-56.5	31	18.9	50	30.5	84	51.5	11	13.9
	M	1.9	5.3	4.3-6.4	55.2	53.1-57.4	14	12.0	26	22.2	48	41.4	14	20.6
Northern Africa (396)	F	5.6	9.3	8.0-10.6	50.8	49.0-52.6	43	21.2	59	29.1	100	49.7	30	29.7
	M	3.5	5.9	4.8-7.0	48.6	47.3-50.0	32	16.6	47	24.3	95	50.3	22	23.4
Sub-Saharan Africa	F	3.0	6.5	3.7-9.3	44.6	41.4-47.9	11	23.4	9	19.1	22	47.8	9	37.5
(114)	M	3.4	4.3	2.1-6.5	44.9	43.2-46.7	12	17.9	9	13.4	31	46.3	10	27.8
Southern Asia (378)	F	4.6	9.7	7.9-11.5	48.8	46.7-50.9	36	26.9	21	15.7	47	35.6	36	42.3
	M	5.8	10.2	8.4-11.9	46.7	45.5-48.0	52	21.3	34	13.9	83	34.3	48	29.8
Rest of Asia (94)	F	1.9	4.5	2.5-6.5	48.4	44.9-51.9	8	17.0	11	23.4	26	55.3	4	19.0
	M	2.3	3.9	2.4-5.4	46.9	43.9-49.8	7	14.9	9	19.1	23	48.9	8	33.3
LA and the Caribbean	F	2.7	4.3	2.2-6.4	51.7	47.5-56.0	4	17.4	5	21.7	9	39.1	4	28.6
(32)	M	3.4	3.2	1.1-5.2	48.6	41.0-56.1	0	0.0	3	33.3	5	55.6	2	50.0

*Excluding those who died or moved in 2010 (n = 302).

**Figure 1:**
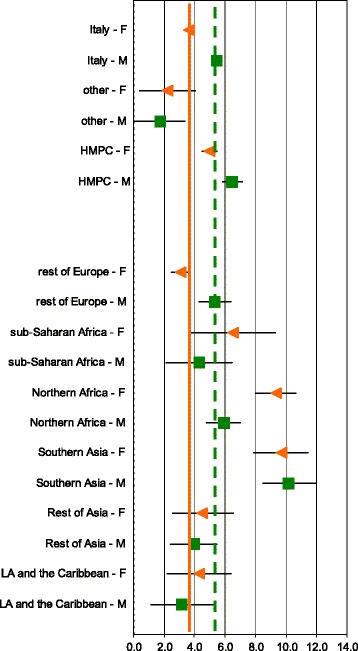
**Age-adjusted prevalence (with 95%CI) by gender and sub-region on December 31** ^**st**^ **, 2009. Age 20–74.** Diabetes register did not include citizens of Oceania and Stateless. Green squares are male prevalence and orange triangles are female prevalence. The green dot axis is the overall male crude prevalence (5.4%) and the orange solid axis is the overall female crude prevalence (3.9%).

For both sexes, citizens of HMPC experienced significantly higher prevalence of diabetes than did Italians (F: 5.0% vs 3.6%; M: 6.5% vs 5.5%), whereas for citizens from HDC, the occurrence of diabetes was smaller (Figure 1 and Table 1). The excess of prevalence in HMPC was mostly due to a strong excess in immigrants from Southern Asia and Northern Africa.

For Italians the prevalence was higher in males than in females, with a similar pattern seen in people from Eastern Europe (Europe no HDC). For Africans, both North and Sub-Saharan, instead, the prevalence was higher in females. People from other areas did not show any difference between sexes.

### Disease management and glycaemic control

The proportion of Italians and of immigrants from HMPC for whom no drug prescription was found was similar, as was the proportion of subjects not followed by diabetes clinics (Table 1).

Instead, the percentage of Italians who did not perform HbA1c test was lower than it was for foreigners. Among HMPC, almost one out of two subjects had not been tested, excluding the citizens from Southern Asia, who had lower proportion of not tested, although still higher than that of Italians.

Ethnic differences were also noticeable when observing percentages of subjects with HbA1c test equal to 9% (75 mmol/mol) or over: for both sexes, the proportion for the HMPC was more than double that for Italians. Broken down into sub-regions, women from Southern Asia and sub-Saharan Africa and men from Latin America had the worst indicators.

The results of multiple logistic regression (Table 2) show differences between Italians and immigrants from HMPC. We did not perform analysis for HDC because the population with diabetes was too small to be investigated.

**Table 2:** **Odds ratios** ^**^**^ **(with 95% CI) for quality of care indicators, age 20–74**

	**Female**		**Male**		**Total**	
**Indicators**	**OR**	**95% CI**	**OR**	**95% CI**	**OR**	**95% CI**
Not in care of diabetes clinics	1.22	0.98-1.50	0.95	0.77-1.17	1.08	0.93-1.25
Without HbA1c test in 2010*	1.84	1.54-2.20	1.29	1.09-1.53	1.51	1.34-1.71
Among tested, HbA1c > =9%						
Adjusted by age	2.32	1.77-3.05	1.94	1.50-2.50	2.12	1.76-2.55
Adjusted by age and treatment	2.38	1.80-3.14	1.82	1.41-2.35	2.06	1.80-3.14

^^^Odds ratios (OR) and 95% confidence intervals (95% CI) were obtained using logistic regression model with age and treatment covariates (for third outcome we performed analysis with and without treatment as covariate). Italy was used as comparator.

*Excluding those who died or moved in 2010 (n = 302).

In comparison with their Italian counterparts, HMPC had a similar odds of not being followed by a diabetes clinic, while both sexes from HMPC had a greater odds of not being tested for HbA1c compared to Italians, as well as of having unsatisfactory control of disease (i.e. HbA1c value equal 9% or over). The results do not substantially change using 7.5% (58 mmol/mol) cut-off [27]: OR adjusted for age, treatment and sex: 1.59; 95% CI: 1.34-1.88.

The outcomes were poorer in female from HMPC for access to diabetes clinics and for HbA1c testing, while there was no difference for HbA1c values (Wald test for heterogeneity p = 0.0850; p = 0.0156; p = 0.6635, respectively).

**Figure 2:**
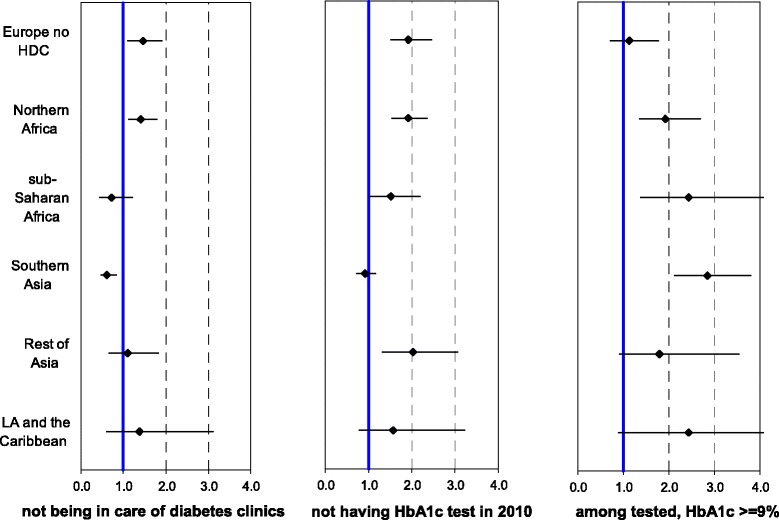
**Adjusted odds ratios (with 95% CI) for quality of care indicators by sub-region on December 31** ^**st**^ **, 2009. Age 20–74.** OR and 95% CI were obtained using multiple logistic regression model with age, treatment, and sex as covariates. Italians were used as reference. OR estimation for Hb1Ac test excludes those who died or moved in 2010 (n = 302).

In the analysis by different sub-regions (Figure 2), we found that subjects from Northern Africa experienced higher odds for all indicators compared to Italians (“not being in care of diabetes clinics”: OR = 1.42; 95% CI: 1.12-1.80; “without HbA1c test in 2010”: OR = 1.91; 95% CI: 1.55-2.35; “among those tested, HbA1c >=9%”: OR = 1.92; 95% CI: 1.37-2.69, respectively).

The Southern Asians seemed to use the diabetes clinic more than did Italians (OR = 0.61; 95% CI: 0.46-0.83), while there was no difference in terms of test execution (OR = 0.92; 95% CI: 0.74-1.15), although the former had worse glycaemic control (OR = 2.84; 95% CI: 2.13-3.79), more markedly so for women than for men (OR = 4.97; 95% CI: 3.06-8.07 and OR = 2.06; 95% CI: 1.42-2.98, data not shown in the figure). Very poor glycaemic control was common to all sub-regions except Europe no HDC.

## Discussion

### Prevalence

Our study used population-based data retrieved from a disease register in a setting with remarkable rates of immigration. We observed a significantly higher prevalence for HMPC citizens than for Italians, and lower prevalence for citizens from HDC countries. The prevalence was particularly high for North Africans and Southern Asians. Our findings are consistent with similar studies conducted in Europe [2,4,7-11], in Canada [3,5], in the USA [28], and in Australia [6]. The high prevalence of diabetes in specific ethnic migrant groups are likely attributable to a complex interplay of genetic and environmental factors, including acculturation, stress, social isolation, and employment and economic challenges [3,29]. In addition, people from Southern Asia seem to have a genetic susceptibility to Type 2 diabetes [30,31]. Furthermore, some authors have suggested that hypovitaminosis D, particularly relevant in migrant groups with darker skin pigmentation, increases the risk of diabetes in Africans [32,33].

While prevalence of diabetes is higher among men than women in Italians, HMPC women in our study had prevalence rates that were roughly equivalent to or higher than those of men from the same countries. Particularly high prevalence was evident among women from Northern Africa compared to men, and, with a smaller gap, among women from sub-Saharan Africa. This pattern, founded previously in Canada, Sweden, and France [2,3,34], suggests that gender plays a role as effect modifier in the relationship between ethnicity and diabetes in non-western countries. A higher prevalence of diabetes in women can be related to a higher rate of obesity and physical inactivity, especially high among women of non-western origin [35,36].

Women from Northern Africa and Southern Asia usually migrate to Italy with their husbands, who seek job opportunities. For example, in 2007, the percentage of residence permits for family reunion issued to Tunisian women was 83.2% and to Indian women 70.1%, while the general percentage was 48.4% [37]. The host environment may reduce the need for physical activity to housework [26] and introduce a more caloric diet, thus contributing to the higher risk of overweight, obesity, and diabetes.

Italian diabetes guidelines recommend initial screening for diabetes at age 45 years [38], although an opportunistic screening at younger age is recommended for patients belonging to an ethnic group at high risk. Our study confirms the rationale of these recommendations and identifies the ethnic groups particularly at risk, as well as North African women and Southern Asians of both sexes.

### Disease management and glycaemic control

Our study did not find any differences between Italians and immigrants in the access of diabetes clinics as a whole, although North Africans proved to use the clinics less than did Southern Asians, who used the clinics more than did Italians. This could be due in part to a general difficulty for immigrants in accessing to primary care, and particularly to the GP or family paediatrician [39]. An Irish study conducted among asylum seekers [40] found that they were more likely to be referred to outpatient services for various medical conditions than were their Irish counterparts.

As regards recent measurement of HbA1c, HMPC were less likely to perform the annual test, the difference being greater for women. Detailed analysis by sub-regions revealed that among HMPC, only Southern Asians had a level of compliance to guidelines similar to that of Italians.

Concerning glycaemic control, HMPC fared worse than did Italians, with the difference once again being more marked for women. For this intermediate outcome, only immigrants from Europe no HDC experienced values similar to those of Italians. Surprisingly, Southern Asians had the worst outcomes in terms of glycaemic control, despite their being the ones who most frequently cared for by specialized diabetes clinics and who seemed to be the most compliant to guidelines for HbA1c testing. Some authors suggest that one important factor contributing to increased Type 2 diabetes in Asian Indians is excessive insulin resistance compared to Caucasians. This difference in the degree of insulin resistance may be explained by either an environmental or a genetic factor or by combination of both [27,28]. Our findings confirm the studies carried out in the UK [13,16,41,42], in Sweden [18], in the USA [15,17], and in Italy [12]. Gray and Heisler did not find any significant difference in frequency of measurement, while Buja underlined less coverage in terms of HbA1c for HMPC compared with Italians. The other studies, focusing on glycaemic control, found worse values for non-white ethnic groups.

Thabit [14] argued in his study that poor levels of health literacy can negatively impact the patients’ ability to interpret blood glucose levels, understand educational materials, and read labels on medication, irrespective of the patient’s educational level when operating in the foreign language. The same author suggested that health knowledge and perceptions about managing diabetes may be conceptualized differently in ethnic minorities compared to the majority population.

A possible explanation may be that, in our setting, immigration is a relatively recent phenomenon and consequently the patients from HMPC on average have been into the care of diabetes clinics for less time compared to Italians. As a result, the higher level of HbA1c could reflect a higher proportion of newly diagnosed and newly treated patients. Nevertheless, it must be noted that all the differences in control of disease have been observed even when we adjusted for treatment.

### Strengths and limitations

In our knowledge, this is the first study carried out in Southern Europe based on diabetes register that is able to determine both age-standardized prevalence and quality of care indicators for immigrants and for different geographical area of origin, using gender approach.

Nevertheless, as the information about diabetes onset is not completely consistent, we decided not include this variable in the analysis. As proxy of disease severity we used diabetes treatment regimen, distinguishing between subjects using diet only and those treated with antidiabetic drugs. In addition, we had no information about the socioeconomic status of diabetic patients or about their BMI values or other clinical characteristics.

## Conclusions

Our study confirms the higher diabetes prevalence in immigrants from HMPC countries, especially for Southern Asian citizens and for women from Northern Africa. When comparing Italians with immigrants from HMPC, although there was almost no difference in access of specialized level of care, the latter were less compliant and more likely to experience worse levels of HbA1c. The failure in disease control is of particular concern, as immigrant patients are quite young and thus their duration of disease will be quite long, thereby leading to potentially more severe diabetes complications.

Our findings highlight the need for special, gender-oriented actions for prevention and early diagnosis of diabetes to contrast the higher risk in Northern Africans and Southern Asians. Further studies are also required to determine whether the protocols in use are adequate for diabetes control but need to be better implemented or whether different protocols and treatment for diabetes in some ethnic groups are needed.

## Additional file

Additional file 1:
**Data by country of origin.**

## Abbreviations

AMD: Italian association of clinical diabetologists
ANOVA: Analysis of variance
CI: Confidence interval
EUPHA: European conference on migrant and ethnic minority health
INMP: Istituto nazionale per la promozione della salute delle popolazioni migranti ed il contrasto delle malattie della povertà
HbA1c: Glycated haemoglobin
HDC: High developed countries
HMPC: High migration pressure countries
HPH: Health promoting hospitals & health services
GP: General practitioner
OR: Odds ratio
TF MFCCH: Task force on migrant friendly and culturally competent health care
UK: United Kingdom
USA: United States of America
