# Supplementary material for: Differences in diabetes prevalence and inequalities in disease management and glycaemic control by immigrant status: a population-based study (Italy)
Source: BMC Public Health. 2015 Feb 6;15:87. doi: 10.1186/s12889-015-1403-4 (PMC4334763; doi:10.1186/s12889-015-1403-4)
Supplement: Additional file 1: — Data by country of origin. [file 12889_2015_1403_MOESM1_ESM.pdf]

Istat code	Country	HDC/HMPC	subregion	population 20-74 years			people with diabetes 20-74 years			crude prevalence (x100)		
				M	F	T	M	F	T	M	F	T
100	Italy	-		162,936	160,740	323,676	9,380	6,509	15,889	5.7569	4.0494	4.9089
701	Australia	HDC		3	2	5						
203	Austria	HDC		7	19	26						
206	Belgium	HDC		14	17	31	1		1	7.1429	0.0000	3.2258
509	Canada	HDC		2	4	6						
212	Denmark	HDC		11	8	19	1		1	9.0909	0.0000	5.2632
214	Finland	HDC		1	18	19						
215	France	HDC		52	92	144		1	1	0.0000	1.0870	0.6944
216	Germany	HDC		46	111	157	1	1	2	2.1739	0.9009	1.2739
220	Greece	HDC		19	6	25						
223	Iceland	HDC			2	2		1	1		50.0000	50.0000
221	Ireland	HDC		3	9	12						
334	Israel	HDC		2	3	5						
326	Japan	HDC		1	15	16						
320	Korea, Democratic People's Republic of	HDC			4	4			0		0.0000	0.0000
232	Netherlands	HDC		15	13	28		1	1	0.0000	7.6923	3.5714
719	New Zealand	HDC			1	1						
231	Norway	HDC		4	3	7		1	1	0.0000	33.3333	14.2857
234	Portugal	HDC		11	16	27						
236	San Marino	HDC		3	4	7						
239	Spain	HDC		27	130	157		1	1	0.0000	0.7692	0.6369
240	Sweden	HDC		4	10	14						
241	Switzerland	HDC		9	9	18						
219	United Kingdom	HDC		57	65	122	1	1	2	1.7544	1.5385	1.6393
536	United States	HDC		11	21	32						
246	Vatican City State	HDC		1		1						
201	Albania	HMPC	Europe no HDC	2,687	2,128	4,815	76	63	139	2.8284	2.9605	2.8868
256	Belarus	HMPC	Europe no HDC	7	63	70						
252	Bosnia and Herzegovina	HMPC	Europe no HDC	49	34	83	3	1	4	6.1224	2.9412	4.8193
209	Bulgaria	HMPC	Europe no HDC	83	130	213	2	2	4	2.4096	1.5385	1.8779
250	Croatia	HMPC	Europe no HDC	61	55	116						
257	Czech Republic	HMPC	Europe no HDC	13	66	79		1	1	0.0000	1.5152	1.2658
247	Estonia	HMPC	Europe no HDC	1	18	19						
244	Hungary	HMPC	Europe no HDC	16	35	51						
272	Kosovo	HMPC	Europe no HDC	16	21	37		1	1	0.0000	4.7619	2.7027
248	Latvia	HMPC	Europe no HDC	12	43	55		1	1	0.0000	2.3256	1.8182
249	Lithuania	HMPC	Europe no HDC	21	45	66						
253	Macedonia (FYROM)	HMPC	Europe no HDC	170	147	317	5	6	11	2.9412	4.0816	3.4700
227	Malta	HMPC	Europe no HDC	1	2	3						
254	Moldova, Republic of	HMPC	Europe no HDC	529	1,277	1,806	9	16	25	1.7013	1.2529	1.3843
270	Montenegro	HMPC	Europe no HDC	1	1	2						
233	Poland	HMPC	Europe no HDC	192	851	1,043	3	10	13	1.5625	1.1751	1.2464
235	Romania	HMPC	Europe no HDC	1,511	2,047	3,558	16	20	36	1.0589	0.9770	1.0118
245	Russian Federation	HMPC	Europe no HDC	46	375	421		2	2	0.0000	0.5333	0.4751
271	Serbia	HMPC	Europe no HDC	377	243	620						
255	Slovakia	HMPC	Europe no HDC	29	44	73						
251	Slovenia	HMPC	Europe no HDC	5	8	13						
243	Ukraine	HMPC	Europe no HDC	464	2,672	3,136	3	41	44	0.6466	1.5344	1.4031
602	Argentina	HMPC	Latin America and the Caribbean	65	83	148	4		4	6.1538	0.0000	2.7027
604	Bolivia, Plurinational State of	HMPC	Latin America and the Caribbean	0	5	5						
605	Brazil	HMPC	Latin America and the Caribbean	132	362	494	2	5	7	1.5152	1.3812	1.4170
606	Chile	HMPC	Latin America and the Caribbean	8	15	23	1		1	12.5000	0.0000	4.3478
608	Colombia	HMPC	Latin America and the Caribbean	65	130	195		5	5	0.0000	3.8462	2.5641
513	Costa Rica	HMPC	Latin America and the Caribbean		4	4						
514	Cuba	HMPC	Latin America and the Caribbean	52	178	230		3	3	0.0000	1.6854	1.3043
515	Dominica	HMPC	Latin America and the Caribbean	2	3	5						
516	Dominican Republic	HMPC	Latin America and the Caribbean	91	206	297	1	8	9	1.0989	3.8835	3.0303
609	Ecuador	HMPC	Latin America and the Caribbean	11	37	48	1	1	2	9.0909	2.7027	4.1667
517	El Salvador	HMPC	Latin America and the Caribbean	6	8	14						
525	Honduras	HMPC	Latin America and the Caribbean	0	2	2						
518	Jamaica	HMPC	Latin America and the Caribbean	1		1						
527	Mexico	HMPC	Latin America and the Caribbean	5	19	24						
529	Nicaragua	HMPC	Latin America and the Caribbean	2	2	4						
530	Panama	HMPC	Latin America and the Caribbean		5	5						
614	Paraguay	HMPC	Latin America and the Caribbean	2	9	11						
615	Peru	HMPC	Latin America and the Caribbean	18	62	80		1	1	0.0000	1.6129	1.2500
618	Uruguay	HMPC	Latin America and the Caribbean	4	10	14						
619	Venezuela, Bolivarian Republic of	HMPC	Latin America and the Caribbean	13	31	44						
401	Algeria	HMPC	Northern Africa	178	96	274	5	3	8	2.8090	3.1250	2.9197
419	Egypt	HMPC	Northern Africa	851	259	1,110	34	12	46	3.9953	4.6332	4.1441
431	Libyan Arab Jamahiriya	HMPC	Northern Africa	6	2	8	1		1	16.6667	0.0000	12.5000
436	Morocco	HMPC	Northern Africa	3,652	3,117	6,769	113	163	276	3.0942	5.2294	4.0774
460	Tunisia	HMPC	Northern Africa	1,245	650	1,895	40	25	65	3.2129	3.8462	3.4301
727	Samoa	HMPC	Oceania		1	1						
725	Solomon Islands	HMPC	Oceania		1	1						
730	Tonga	HMPC	Oceania	2		2						
301	Afghanistan	HMPC	Rest of Asia	1		1						
358	Armenia	HMPC	Rest of Asia	1	1	2						
359	Azerbaijan	HMPC	Rest of Asia	1	1	2						
314	China	HMPC	Rest of Asia	1,496	1,483	2,979	32	22	54	2.1390	1.4835	1.8127
360	Georgia	HMPC	Rest of Asia	50	293	343	1	4	5	2.0000	1.3652	1.4577
331	Indonesia	HMPC	Rest of Asia	2	13	15						
333	Iraq	HMPC	Rest of Asia	1	3	4						
327	Jordan	HMPC	Rest of Asia	13	8	21		2	2	0.0000	25.0000	9.5238
356	Kazakhstan	HMPC	Rest of Asia	1	11	12		1	1	0.0000	9.0909	8.3333
335	Kuwait	HMPC	Rest of Asia	1		1						
361	Kyrgyzstan	HMPC	Rest of Asia		6	6						
336	Lao People's Democratic Republic	HMPC	Rest of Asia	7	4	11	1		1	14.2857	0.0000	9.0909
337	Lebanon	HMPC	Rest of Asia	8	8	16						
340	Malaysia	HMPC	Rest of Asia		2	2						
323	Philippines	HMPC	Rest of Asia	115	217	332	6	8	14	5.2174	3.6866	4.2169
346	Singapore	HMPC	Rest of Asia	1		1						
348	Syrian Arab Republic	HMPC	Rest of Asia	8	10	18	1	1	2	12.5000	10.0000	11.1111
363	Taiwan, Province of China	HMPC	Rest of Asia	3	5	8						
349	Thailand	HMPC	Rest of Asia	4	77	81						
351	Turkey	HMPC	Rest of Asia	334	247	581	4	8	12	1.1976	3.2389	2.0654
357	Uzbekistan	HMPC	Rest of Asia	8	31	39						
353	Viet Nam	HMPC	Rest of Asia	18	26	44	1	1	2	5.5556	3.8462	4.5455
354	Yemen	HMPC	Rest of Asia	1	4	5	1		1	100.0000	0.0000	20.0000
305	Bangladesh	HMPC	Southern Asia	51	26	77	3	2	5	5.8824	7.6923	6.4935
330	India	HMPC	Southern Asia	2,335	1,735	4,070	122	72	194	5.2248	4.1499	4.7666
332	Iran, Islamic Republic of	HMPC	Southern Asia	8	6	14						
342	Nepal	HMPC	Southern Asia	2	3	5						
344	Pakistan	HMPC	Southern Asia	1,853	861	2,714	92	42	134	4.9649	4.8780	4.9374
311	Sri Lanka	HMPC	Southern Asia	505	307	812	27	18	45	5.3465	5.8632	5.5419
402	Angola	HMPC	sub-Saharan Africa	6	11	17						
406	Benin	HMPC	sub-Saharan Africa	14	10	24		1	1	0.0000	10.0000	4.1667
409	Burkina Faso	HMPC	sub-Saharan Africa	186	87	273	1	3	4	0.5376	3.4483	1.4652
410	Burundi	HMPC	sub-Saharan Africa	3		3						
411	Cameroon	HMPC	sub-Saharan Africa	31	43	74	1		1	3.2258	0.0000	1.3514
413	Cape Verde	HMPC	sub-Saharan Africa	3	7	10						
414	Central African Republic	HMPC	sub-Saharan Africa	2		2				0.0000		0.0000
415	Chad	HMPC	sub-Saharan Africa		1	1					0.0000	0.0000
418	Congo	HMPC	sub-Saharan Africa	9	7	16		1	1	0.0000	14.2857	6.2500
463	Congo, The Democratic Republic of The	HMPC	sub-Saharan Africa	15	11	26		1	1	0.0000	9.0909	3.8462
404	Côte D'Ivoire	HMPC	sub-Saharan Africa	114	71	185	2		2	1.7544	0.0000	1.0811
424	Djibouti	HMPC	sub-Saharan Africa		1	1		1	1		100.0000	100.0000
427	Equatorial Guinea	HMPC	sub-Saharan Africa		1	1						
466	Eritrea	HMPC	sub-Saharan Africa	17	14	31						
420	Ethiopia	HMPC	sub-Saharan Africa	16	32	48						
421	Gabon	HMPC	sub-Saharan Africa	1		1						
422	Gambia	HMPC	sub-Saharan Africa	4	2	6	1		1	25.0000	0.0000	16.6667
423	Ghana	HMPC	sub-Saharan Africa	1,058	756	1,814	31	24	55	2.9301	3.1746	3.0320
425	Guinea	HMPC	sub-Saharan Africa	58	32	90						
426	Guinea-Bissau	HMPC	sub-Saharan Africa	1		1						
428	Kenya	HMPC	sub-Saharan Africa	2	4	6						
429	Lesotho	HMPC	sub-Saharan Africa		1	1						
430	Liberia	HMPC	sub-Saharan Africa	61	6	67						
432	Madagascar	HMPC	sub-Saharan Africa	2	1	3						
435	Mali	HMPC	sub-Saharan Africa	14	4	18						
437	Mauritania	HMPC	sub-Saharan Africa	2	1	3						
438	Mauritius	HMPC	sub-Saharan Africa	36	38	74	4	1	5	11.1111	2.6316	6.7568
440	Mozambique	HMPC	sub-Saharan Africa	9	1	10						
442	Niger	HMPC	sub-Saharan Africa	8	6	14	1		1	12.5000	0.0000	7.1429
443	Nigeria	HMPC	sub-Saharan Africa	338	474	812	12	14	26	3.5503	2.9536	3.2020
446	Rwanda	HMPC	sub-Saharan Africa	3	1	4						
449	Sao Tome and Principe	HMPC	sub-Saharan Africa	1		1						
450	Senegal	HMPC	sub-Saharan Africa	435	158	593	12	1	13	2.7586	0.6329	2.1922
451	Sierra Leone	HMPC	sub-Saharan Africa	8		8						
453	Somalia	HMPC	sub-Saharan Africa	27	21	48	1		1	3.7037	0.0000	2.0833
454	South Africa	HMPC	sub-Saharan Africa	4	2	6						
455	Sudan	HMPC	sub-Saharan Africa	13		13						
456	Swaziland	HMPC	sub-Saharan Africa	1		1						
458	Togo	HMPC	sub-Saharan Africa	20	11	31	1		1	5.0000	0.0000	3.2258
461	Uganda	HMPC	sub-Saharan Africa		1	1						
999	Stateless	HMPC		13	3	16						
		Total		185,304	184,131	369,435	10,061	7,134	17,195	5.43	3.87	4.65
